# Acute granulomatous iridocyclitis in a child with tubulointerstitial nephritis and uveitis syndrome

**DOI:** 10.1186/s12348-015-0035-2

**Published:** 2015-02-13

**Authors:** Kenan Barut, Turkay Rzayev, Nur Canpolat, Yasemin Ozluk, Ilknur Tugal-Tutkun, Ozgur Kasapcopur

**Affiliations:** Department of Pediatric Rheumatology, Cerrahpasa Faculty of Medicine, Istanbul University, Istanbul, Turkey; Department of Pediatric Nephrology, Cerrahpasa Faculty of Medicine, Istanbul University, Istanbul, Turkey; Department of Pathology, Istanbul Faculty of Medicine, Istanbul University, Istanbul, Turkey; Department of Ophthalmology, Istanbul Faculty of Medicine, Istanbul University, Capa, 34093 Istanbul, Turkey

**Keywords:** Tubulointerstitial nephritis, TINU syndrome, Uveitis, Granulomatous iridocyclitis

## Abstract

**Background:**

Tubulointerstitial nephritis and uveitis [TINU] syndrome is a rare disorder that may also be underdiagnosed. Patients with TINU syndrome typically present with an acute bilateral nongranulomatous anterior uveitis following symptoms of systemic illness.

**Findings:**

We report the case of a 15-year-old girl who presented with acute granulomatous iridocyclitis and was diagnosed with TINU syndrome based on renal biopsy findings. Both her uveitis and interstitial nephritis promptly responded to high-dose corticosteroid treatment, and there were no relapses during follow-up of 20 months.

**Conclusions:**

TINU should be included in the differential diagnosis of patients who present with acute granulomatous uveitis.

## Findings

### Introduction

Tubulointerstitial nephritis and uveitis [TINU] syndrome is a rare disorder, first described in 1975 [1]. It accounts for less than 2% of all uveitis cases, but 10% of cases of acute bilateral anterior uveitis [2]. The diagnosis of TINU syndrome is based on the presence of both acute interstitial nephritis and uveitis in the absence of any other disease that can cause either manifestations [3]. A definitive diagnosis requires the finding of consistent histopathologic changes on renal biopsy specimens [3]. Patients with TINU syndrome typically present with an acute-onset bilateral nongranulomatous anterior uveitis. [3,4] Herein, we report the case of a 15-year-old female who presented with acute granulomatous iridocyclitis associated with constitutional symptoms and who was diagnosed as TINU syndrome based on renal biopsy findings.

### Case report

**Figure 1:**
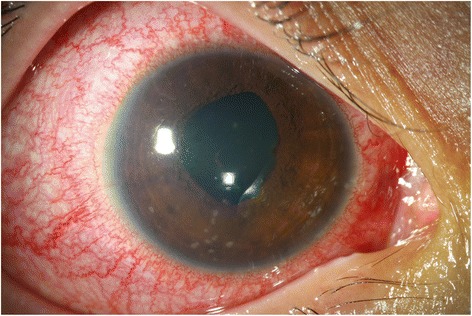
**Slit-lamp photograph with TINU syndrome showing ciliary injection, granulomatous keratic precipitates, and an irregular pupil.**

**Figure 2:**
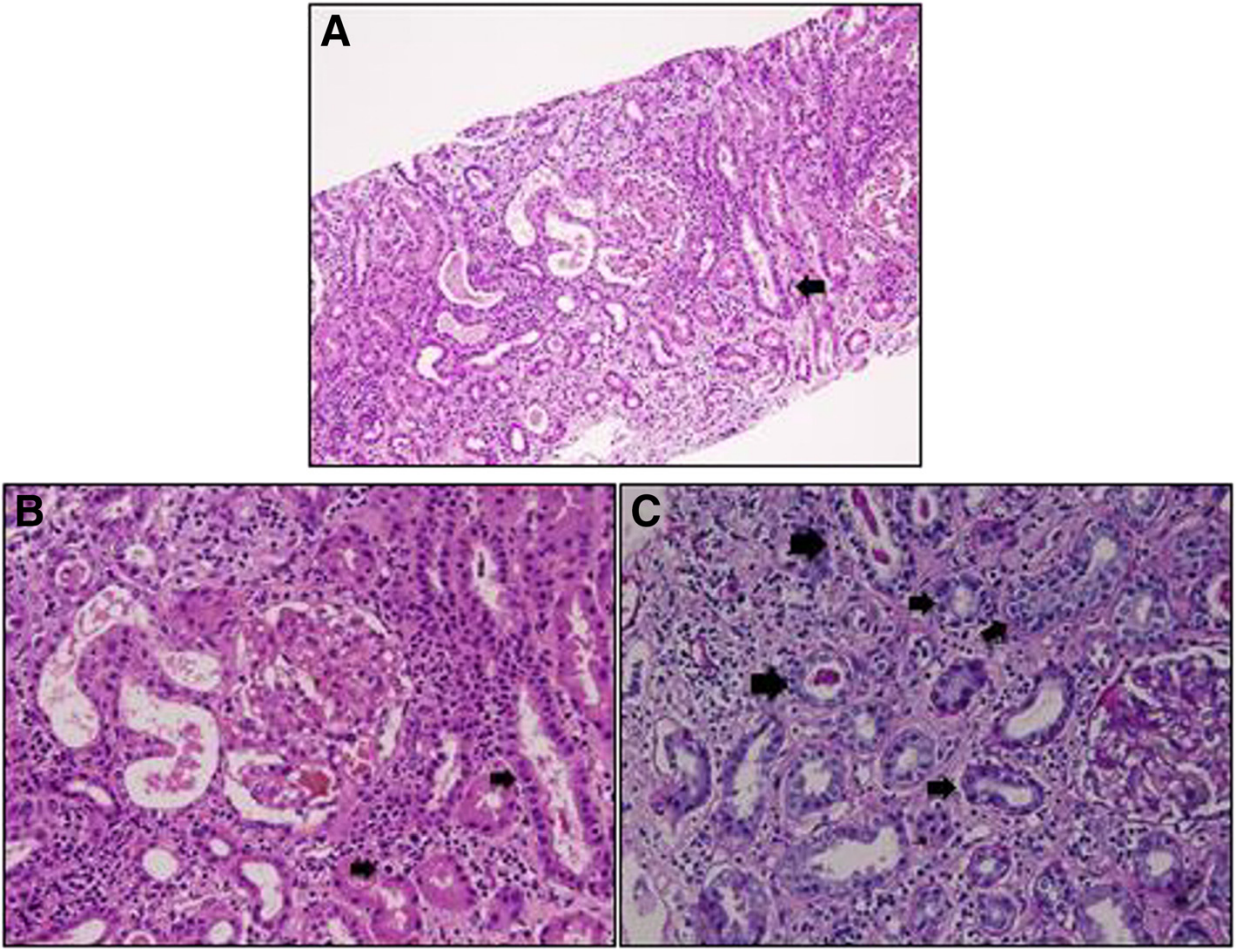
**Interstitial mixed inflammatory infiltration, tubulitis, and focal granular debris within tubular lumina.** Interstitial mixed inflammatory infiltration, tubulitis [small arrows] and focal granular debris within tubular lumina [large arrows]. **(A)** HE ×100, **(B)** HE ×200x. **(C)** PAS ×200 in the renal biopsy specimen of patient with TINU syndrome.

A 15-year-old female was admitted with a 20-day history of extreme fatigue, loss of appetite, and weight loss (7 kg in 1 month). She also had red eyes and decreased vision for 10 days. Her past medical history, as well as family history, were unremarkable. There was no history of any drug use. On physical examination at admission, her weight was 47 kg (3 to 10 percentile), height was 162 cm (50 to 75 percentile), and her blood pressure was 110/70 mmHg. Other physical examination findings were normal. Her visual acuity was 0.4 in the right and 1.0 in the left eye. Slit-lamp examination revealed a marked diffuse ciliary injection, mild corneal edema, large granulomatous keratic precipitates distributed in the Arlt's triangle, 4+ cells in the anterior chamber, Busacca nodules, posterior synechiae, and 0.5+ cells in the anterior vitreous in the right eye (Figure 1). There were fine keratic precipitates, 3+ cells in the anterior chamber, mobile pupil, and 0.5+ cells in the anterior vitreous in the left eye. Anterior chamber flare measured by laser flare photometry was 378 ph/ms in the right eye and 11 ph/ms in the left eye. Intraocular pressure was 10 mmHg in the right and 14 mmHg in the left eye. There was no vitreous haze and the fundus was normal in both eyes. Intensive topical corticosteroid therapy was started. Her laboratory findings suggested mild renal insufficiency findings: serum urea was 76 mg/dl, creatinine level was 1.2 mg/dl (glomerular filtration rate (GFR) 55.8 ml/min/1.73 m^2^) both of which were elevated. The patient's erythrocyte sedimentation rate [112 mm/h] and C-reactive protein (CRP) level (2 mg/dl) were increased. Complete blood count was normal. Antinuclear antibodies were positive in speckled pattern. Anti-ds-DNA (35.45 U/ml) was elevated (normal value <25). C3 (1.5 gr/l), and C4 (0.28 gr/l) levels were found to be normal. Urinalysis showed low urine density (1005), normoglycemic glycosuria (+++, urine dipstick), and nonnephrotic proteinuria (+, urine dipstick). Spot urine protein level was 112.7 mg/dl; urinary creatinine level was 75.5 mg/dl. We found a high protein to creatinine ratio which was 0.67 (*n* < 0.2) and high urinary β2-microglobulin levels (45.3 mg/l, normal values 0.02 to 0.25 mg/l). Urinary tract ultrasound examination was normal. Viral serology was noncontributory. QuantiFERON test was done to rule out tuberculosis as a possible cause of granulomatous uveitis, which was found to be negative. Chest X-ray was normal; angiotensin-converting enzyme (ACE) and lysozyme levels were normal. A renal biopsy was performed. The biopsy specimen showed dense lymphocytes, plasmocytes, and variable eosinophiles in the interstitium and tubulitis in the tubule as well as focal debris and hyaline cylinders in the tubule (Figure 2). Glomerular structures were preserved. There were no vascular lesions. There was no immune complex nephritis. These findings were consistent with acute tubulointerstitial nephritis, and the patient was diagnosed as TINU syndrome. The patient received 2 mg/kg of prednisone for 3 weeks, and then the dose was tapered and discontinued within 1 month. Her kidney function normalized after prednisone therapy. After about 3 weeks of treatment, sedimentation rate (24 mm/h) and CRP level (0.11 mg/dl) decreased. Urea (39 mg/dl) and creatinine (0.9 mg/dl) (GFR 74.3 ml/min/1.73 m^2^) levels returned to normal. Urine analysis, proteinuria, and glycosuria improved rapidly. Uveitis promptly responded to systemic and local corticosteroid treatment, and visual acuity increased to 1.0 within 6 days in the right eye. There were no relapses of uveitis or ocular complications during follow-up of 20 months.

## Discussion

Clinical presentation of TINU is variable. Systemic symptoms such as weight loss, fatigue, arthralgia, and fever may predominate. Renal manifestations include sterile pyuria, hematuria, subnephrotic proteinuria, and renal insufficiency. Ocular symptoms can precede [21%] or follow [65%] the renal diagnosis [3]. Our patient had ocular symptoms, weight loss, fatigue, and loss of appetite. Mild renal insufficiency was present. There is no single diagnostic test available for TINU syndrome. Diagnosis is based on the exclusion of systemic diseases such as Wegener's granulomatosis, systemic lupus erythematosus, Sjogren's syndrome, sarcoidosis, rheumatoid arthritis, Behçet disease, tuberculosis, and brucellosis. Our patient's clinical and biochemical features were compatible with renal failure of tubular origin (increased β2-microglobulin levels, microalbuminuria, glycosuria, and low urine osmolality), without any sign of significant glomerular involvement (normal serum immunoglobulins and complement and nonnephrotic proteinuria). Renal biopsy confirmed acute tubulointerstitial nephritis with inflammatory tubulointerstitial involvement that spared glomerular structures. Goda and colleagues [5] noted that in 11 of 12 patients with TINU syndrome, urinary β2-microglobulin was increased. We also detected increased β2-microglobulin levels in our case.

Ocular involvement in TINU syndrome is in the form of nongranulomatous anterior uveitis in 80% of patients but may also manifest as intermediate, posterior, or panuveitis [3]. Ali and Rosenbaum [6] have recently suggested that sarcoidosis and TINU may have a common pathogenesis based on their observation of inferiorly located chorioretinal scars in four patients with TINU. An anterior uveitis with granulomatous keratic precipitates or iris nodules has been only rarely reported in TINU patients [3]. Our case presented with a severe granulomatous anterior uveitis which may be considered as a more suggestive sign of ocular sarcoidosis. A definitive diagnosis of TINU could be reached by further investigations including renal biopsy.

Our patient responded to the use of high-dose corticosteroid therapy. Her kidney function normalized, and uveitis resolved shortly after induction of treatment. No recurrence of either nephritis or uveitis was observed during tapering or after discontinuation of treatment. However, recurrences and relapses of uveitis have been reported in TINU patients and steroid-sparing immunosuppressive therapy may be required [3].

In conclusion, TINU syndrome is probably an underdiagnosed disorder and should be considered in the differential diagnosis of granulomatous uveitis as well. Diagnostic work-up should include urinalysis and kidney function tests in patients with an acute-onset uveitis with nongranulomatous or granulomatous features. A renal biopsy may be required to make a definitive diagnosis and to exclude confounding entities especially in patients presenting with atypical ocular lesions.

## Consent

Written informed consent was obtained from the patient and her parents for the publication of this report and any accompanying images.

## Abbreviations

TINU: Tubulointerstitial nephritis and uveitis
ph/ms: Photons per millisecond
GFR: Glomerular filtration rate
CRP: C-reactive protein
ACE: Angiotensin-converting enzyme
